# Two Cases of Enlarged Zuckerkandl's Tubercle of the Thyroid Displacing the Recurrent Laryngeal Nerve Laterally

**DOI:** 10.1155/2011/303861

**Published:** 2011-07-10

**Authors:** Emin Gurleyik

**Affiliations:** Department of Surgery, Medical Faculty, Duzce University, 81620 Düzce, Turkey

## Abstract

The thyroid has many anatomic variations. Zuckerkandl's tubercle (ZT) is the posterior extension of lateral lobes. ZT has a relation with the recurrent laryngeal nerve (RLN). RLN lateral to ZT is an uncommon occurrence. This paper presents two cases of this uncommon situation. A 60-year-old female patient with large multinodular goiter was treated with total thyroidectomy. A 69-year-old male patient with follicular neoplasm was treated with total lobectomy. The inferior thyroid arteries and the recurrent laryngeal nerves were identified with usual lateral approach. A left ZT was found in case 1 and a right ZT in case 2. Distal parts of the RLNs were displaced laterally by enlarged ZTs in both cases. Grade 3 ZTs composed of thyroid tissue were placed between the trachea and the RLNs. The ZT is a common anatomic feature of the thyroid. Close relation of the tubercle with the RLN is an important surgical entity. The enlarged ZT seldom pushes the nerve laterally. The knowledge of the anatomy of ZT and its relation with the RLN including all variations is mandatory for safe thyroid operations.

## 1. Introduction

Surgical procedures on the thyroid gland constitute greater part of endocrine surgery. The thyroid has a lot of embryologic, anatomic, and topographic variations affecting the safety of surgical operations. Therefore, a thyroid surgeon must have intimate knowledge of all structural and anatomic variants of the gland. Emil Zuckerkandl (1849–1910), an Austrian anatomist who described a lateral projection of the thyroid gland, was an early pioneer of clinical anatomy [1]. Zuckerkandl's tubercle (ZT) is defined as the extension of the lateral lobes of the thyroid, composing of thyroid tissue only [2]. This has a relationship with the recurrent laryngeal nerve (RLN) at its distal part. The close association of RLN to an enlarged ZT has been documented in many patients [3]. An enlarged ZT (grade 3) displacing RLN may increase injury risk to the nerve during total thyroidectomy. 

 In this case report, we present two patients with nodular goiter accompanied with enlarged ZT displacing RLNs laterally.

## 2. Cases Report

**Case 1**

A 60-year-old female patient with large goiter has been presented to our department with a long-term history of thyroid gland enlargement.

Physical examination, blood chemistry for TSH and FT4, and ultrasound imaging established the diagnosis of euthyroid multinodular goiter. Thyroid ultrasound showed multiple hypoechoic solid nodules in both two lobes and isthmus of the gland. The larger nodules were 32 × 20, 21 × 14, and 22 × 18 mm with irregular margins. The patient was treated with total thyroidectomy.

**Case 2**

A 69-year-old man with nodular goiter was evaluated in our department. Thyroid ultrasound showed a 21 × 20 × 23 mm isoechoic nodule in the right lobe and a 6 mm nodule in the left lobe. After fine needle aspiration from dominant nodule, cytological diagnosis was follicular neoplasm. The patient was treated with total “diagnostic” right lobectomy. Final pathologic diagnosis was follicular adenoma.

**Figure 1:**
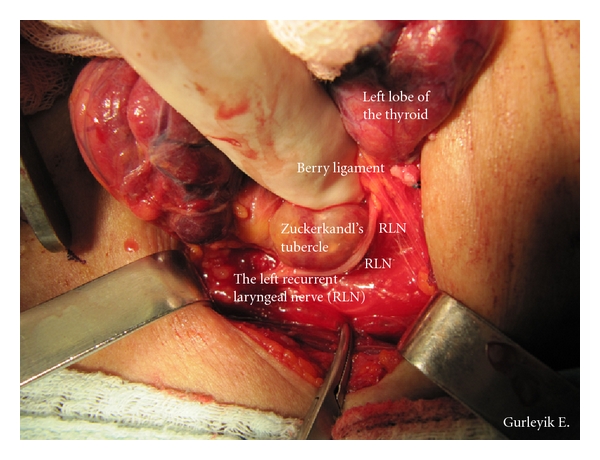
Enlarged tubercle of Zuckerkandl (Case 1) placed between the trachea and the left RLN. The distal part of the RLN was displaced laterally.

**Figure 2:**
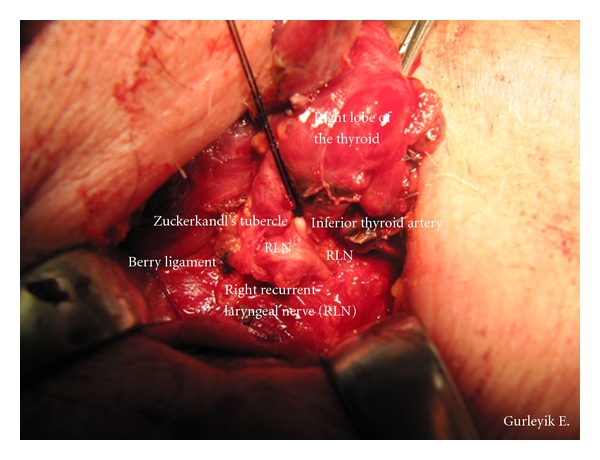
A grade 3 Zuckerkandl's tubercle (Case 2) displaced the right RLN laterally.

**Figure 3:**
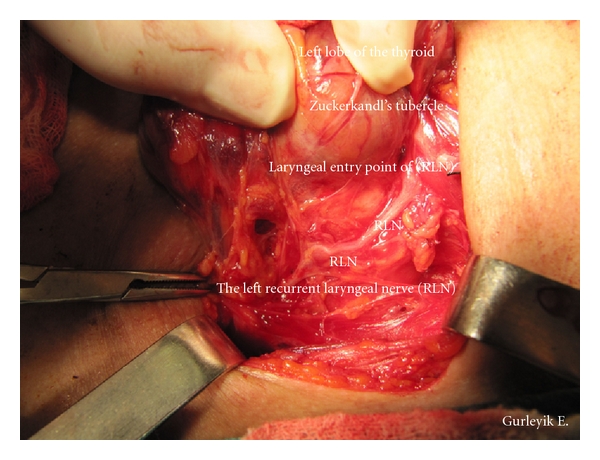
After freeing the tubercle of Zuckerkandl and Berry ligament, the RLN returned back to its usual position in trachea-esophageal groove.

The inferior thyroid arteries were identified, and isolated, and a loop of silk suture was placed around arteries for traction. With the usual lateral approach, RLNs were identified below the artery and fully isolated in both sides of Case 1 and in the right side of Case 2 after freeing and medially mobilizing the thyroid gland. RLNs were explored at anticipated crossing point of the nerves and the arteries. The dissection was advanced upward direction through the Berry ligament. The identified RLNs were followed towards their laryngeal entry points. Distal parts of the left RLN in Case 1 and the right RLN in Case 2 were located more laterally than the usual position. We observed that distal parts of these RLNs were displaced laterally by posterior extensions of the thyroid lobes (Figures 1 and 2). The enlarged ZTs were placed between the trachea and the RLNs. After freeing the tubercles and excising the Berry ligament (posterior suspensor ligament of the gland), the RLNs returned back into trachea-esophageal groove (Figure 3). Excision of this ligament permits easy medial mobilisation of the gland, and the traction on RLN is released in order to return the nerve to its normal anatomic position. These were two ZTs of grade 3. Pathological examination of the tubercles showed that they were composed of thyroid tissue.

## 3. Discussion

 The safety of thyroid operations mainly depends on complete knowledge of the embryology, anatomy, and topography of the thyroid gland and related vascular, nervous structures and parathyroid glands including all potential variations. Delbridge [4] pointed out that completeness of thyroid resection requires the awareness of and attention to pyramidal remnants, to abnormalities associated with the ZT, and to thyrothymic thyroid rest. The ZT is a poorly known and variable anatomic feature of the thyroid gland which maybe not, in fact, so rare [5]. Page et al. [5] proposed that the tubercle should be included in the “nomina anatomica” as the processus posterior glandulae thyroidea described by Zuckerkandl. It is classified into three grades according to size: grade 1: <0.5 cm, grade 2: 0.5 < 1 cm, and grade 3: >1 cm [6, 7]. Based on this classification, our patients have grade 3 tubercles formation changing surgical anatomy of the gland and the RLN.

 The relationship between RLN and ZT has been reported in order to prevent surgical injury to the nerve. Identification and preservation of the RLN is a major concern during thyroidectomies. The knowledge of the lobe of Zuckerkandl is essential to perform safe thyroidectomy without injury for the vascular and nervous structures [2, 6, 7]. Our observation during surgical dissection supported this situation that ZTs had close relationship with the nerve. In our patients, the enlarged ZTs have been situated between the trachea and the RLN which were pushed laterally and superficially by the ZTs. Page et al. [5] identified five (7%) ZTs in 79 patients. On the other hand, Gauger et al. [8] identified ZT in 63% of patients (grade 3 in 45%) of whom the RLN was found lateral to the ZT in only 7% of patients. Our cases are also good examples of this uncommon topographic relation which renders the nerve more vulnerable. The distal part of RLN returned back to its usual position after careful dissection for freeing ZT and Berry ligament. The site of greatest risk during thyroidectomy to the RLN is in the 2-3 cm course of the nerve above the trunk of the inferior thyroid artery where tension forms an artificial genu of the nerve [9]. The presence of a grade 3 ZT may increase the risk of injury in this dangerous site, especially with the situation of the tubercle pushing the nerve laterally. 

 The ZT is a poorly known but a common anatomic feature of the thyroid gland. Resection of the enlarged tubercle is indispensable for completeness of thyroidectomy. The close relation of the tubercle with the RLN is an important surgical entity. The enlarged ZT seldom pushes the nerve laterally and superficially. The knowledge of the anatomy of ZT and its relation with the RLN including all the anatomic and topographic variations is mandatory for safe thyroid operations.
